# Supplementary material for: A ‘parameiosis’ drives depolyploidization and homologous recombination in Candida albicans
Source: Nat Commun. 2019 Sep 26;10:4388. doi: 10.1038/s41467-019-12376-2 (PMC6763455; doi:10.1038/s41467-019-12376-2)
Supplement: Supplementary file 1 — Supplementary Information [file 41467_2019_12376_MOESM1_ESM.pdf]

Supplementary Information

A ‘Parameiosis’ Drives Depolyploidization and Homologous Recombination in *Candida albicans*

Anderson, *et al*.,

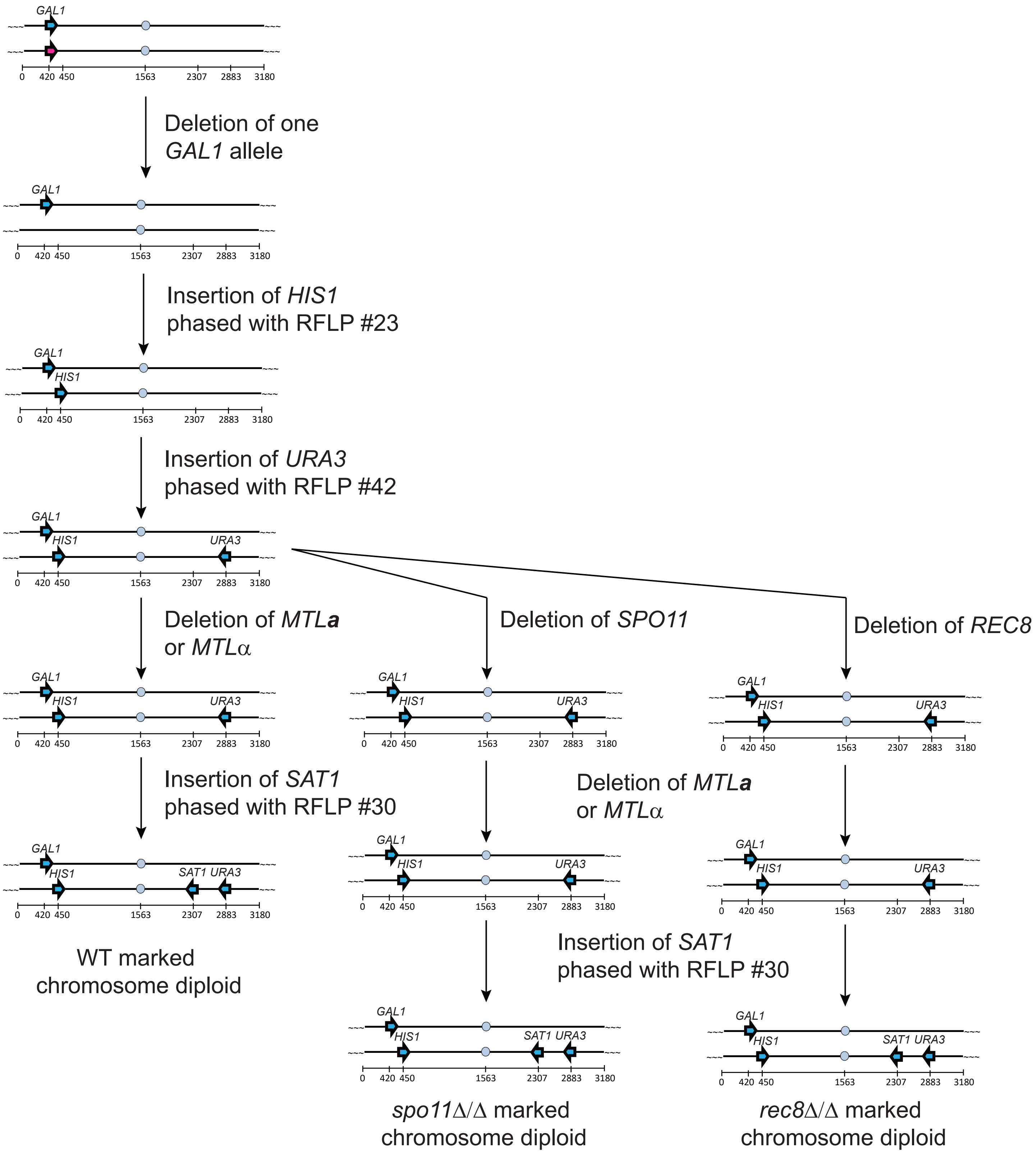

Supplementary Figure 1. Schematic for construction of genetically marked strains. Outline of strain construction to integrate phased markers across Chr1 for all strain backgrounds. The RFLP positions denoted for integration of each marker gene and assignment to a specific homolog correspond to previously annotated SNP-RFLP positions in SC5314.

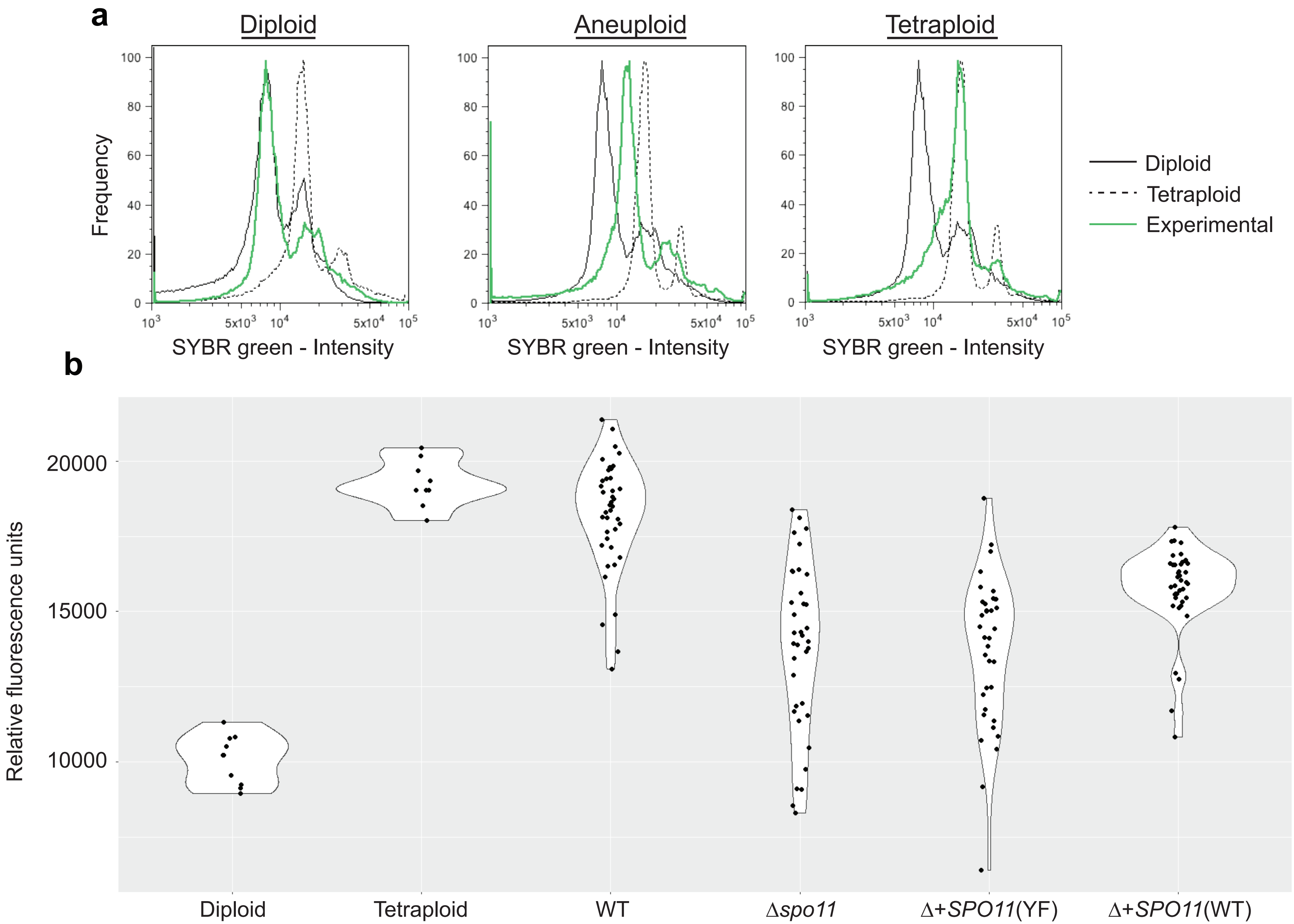

Supplementary Figure 2. DNA quantification of CCL products across *SPO11* genotypes. (**a**) Flow cytometry of control diploid and tetraploid cells along with an aneuploid sample. The two peaks correspond to cells in G1 and G2, whereas DNA for aneuploid populations (green line) resides between the diploid (solid black line) and tetraploid (dashed black line) controls. (**b**) Cells were cultured on PRE-SPO medium for 7 days at 37°C and then plated to single colonies on SCD medium. Forty colonies from each plate were grown overnight in YPD liquid media and the genomic content determined by flow cytometry of SYBR green stained DNA. The relative fluorescent units were measured as the mean of the G1 peak. *n* = 36 biologically independent samples.

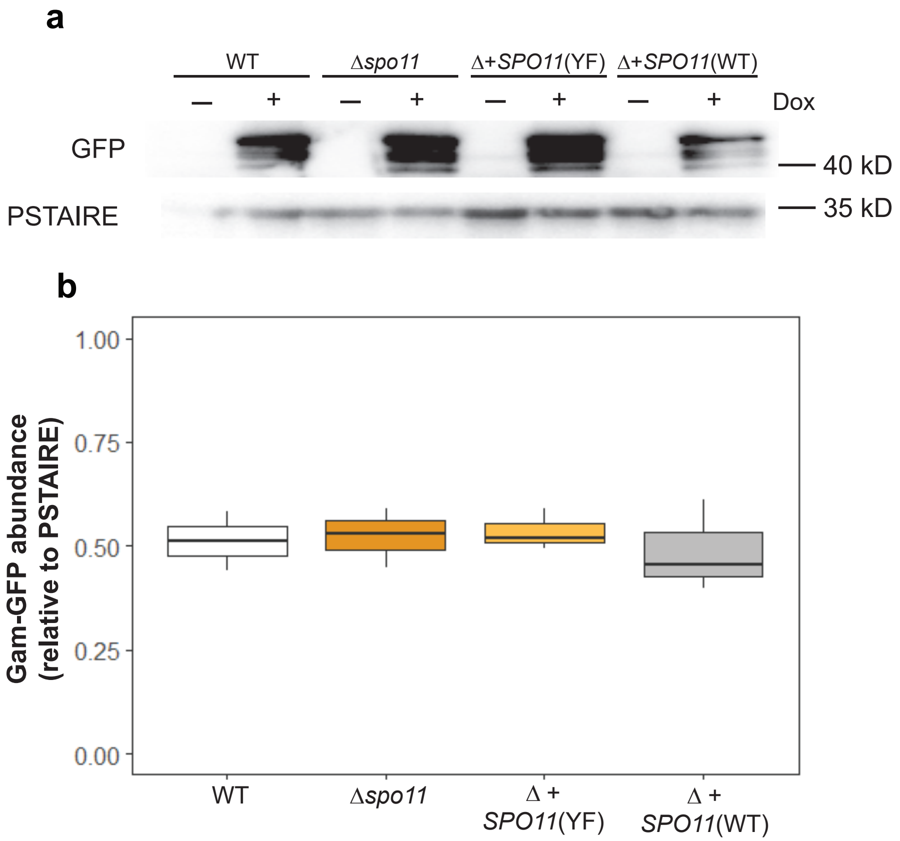

Supplementary Figure 3. Regulation of Gam-GFP expression in C. albicans. (**a**) Cell lysates were collected from strains encoding Gam-GFP in the absence or presence of 50 μg/mL doxycycline. Lysates were probed for GFP to indicate Gam expression using PSTAIRE quantification as a loading control. (**b**) Protein abundance of Gam-GFP was quantified by densitometry of Western blots for Gam-GFP compared to the loading control across *SPO11* genotypes. Three biological replicates were performed for each genotype. Scale bars = 5 μm. White, orange, light orange, and grey denote WT, Δ*spo11*, Δ+*SPO11*(YF), and Δ+*SPO11*(WT), respectively.

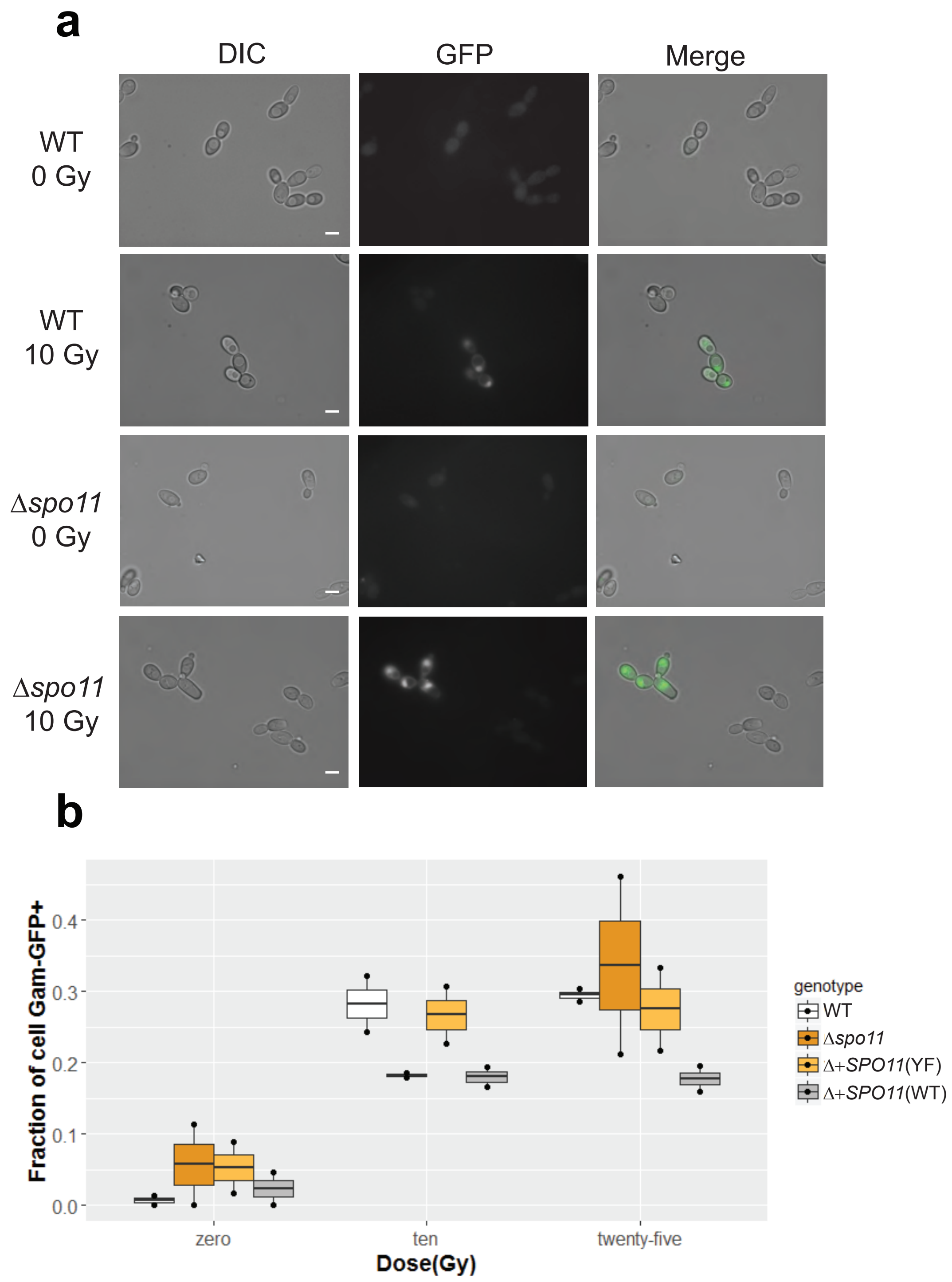

Supplementary Figure 4. Induction of DNA breaks by gamma irradiation. (**a**) Wildtype and Δ*spo11* tetraploid cells encoding Gam-GFP were exposed to 0, 10, and 25 grays (Gy) of gamma irradiation following overnight growth in 50 μg/mL doxycycline. Cells exposed to ionizing radiation displayed fluorescence signal. (**b**) The fraction of Gam-GFP positive cells across five fields of view was quantified across the three radiation doses. Two biological replicates were performed for each strain at each dose. Boxplots are presented as the 75th to 25th percentile with the thick line denoting the median. Whiskers indicate the largest and smallest values within 1.5x of the interquartile range. White, orange, light orange, and grey denote WT, Δ*spo11*, Δ+*SPO11*(YF), and Δ+*SPO11*(WT), respectively.

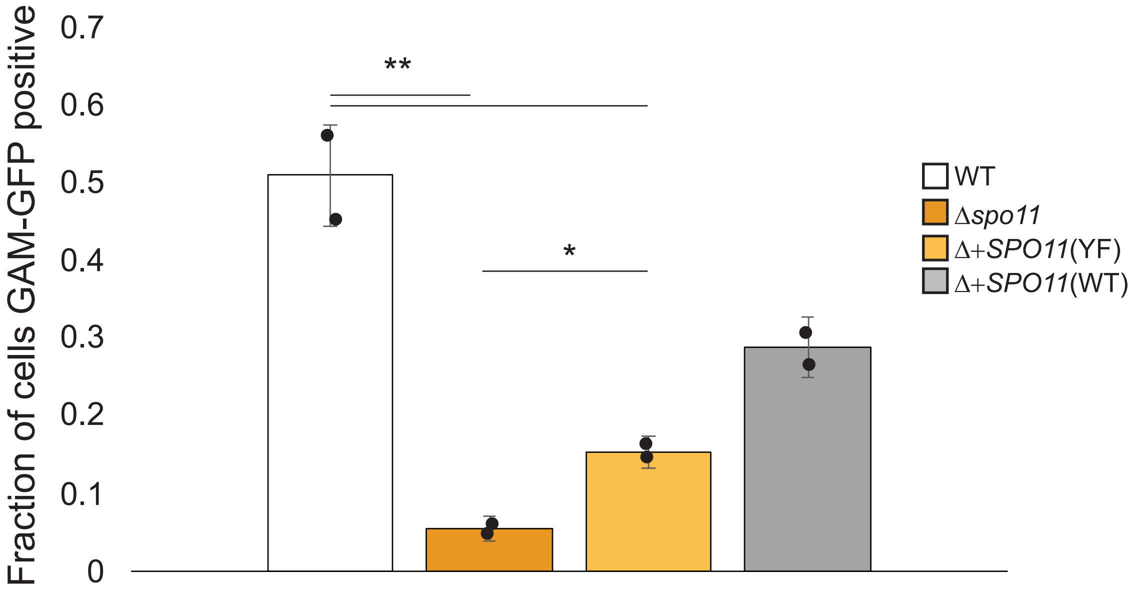

Supplementary Figure 5. Frequency of tetraploid cells expressing Gam-GFP in different *SPO11* backgrounds during CCL. The fraction of cells containing Gam-GFP signal across 10 separate fields was quantified for two strains of each genotype.* denotes $p<0.05$. ** denotes $p<0.01$ by two sample t-test. Error bars indicate standard deviation. White, orange, light orange, and grey denote WT, Δ*spo11*, Δ+*SPO11*(YF), and Δ+*SPO11*(WT), respectively. Two biological replicates were performed for each genotype.

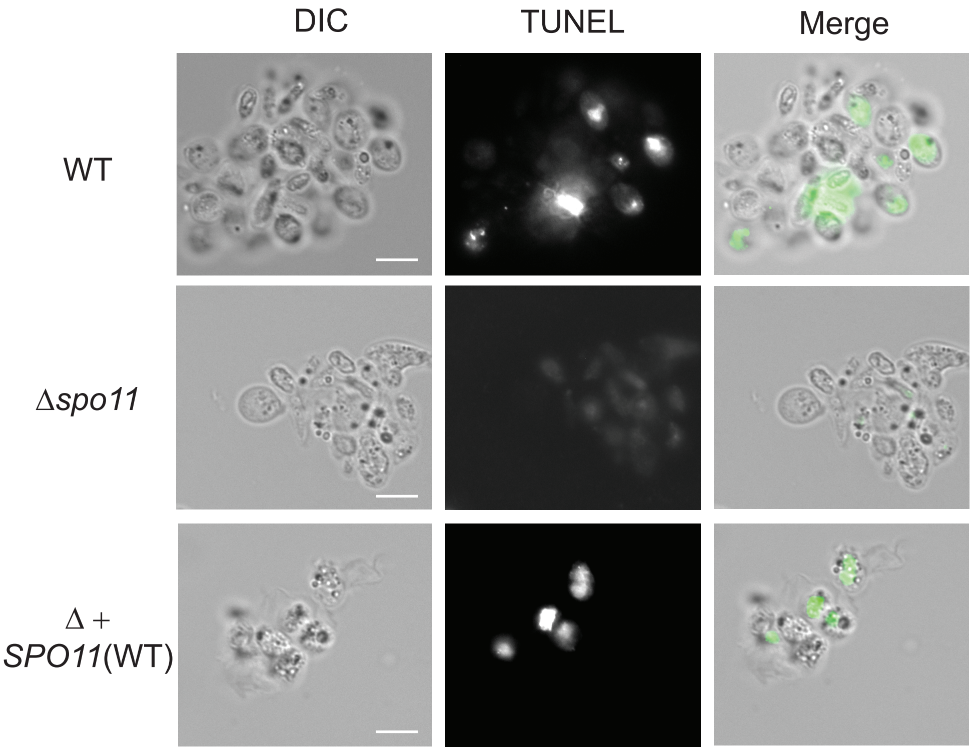

Supplementary Figure 6. TUNEL staining of *C. albicans* cells undergoing CCL. Cells were grown on PRE-SPO agar medium at 37°C to induce CCL for 48 h, fixed in formaldehyde, and spheroplasted with zymolyase. DSB formation in spheroplasted cells was detected using a TUNEL assay. Scale bars = 5 μm.

```
C. albicans_A   ------------------------------------------------------------
C. albicans_B   ------------------------------------------------------------
S. cerevisiae   MALEGLRKKYKTRQELVKALTPKRRSIHLNSNGHSNGTPCSNADVLAHIKHFLSLAANSL

allele_a        -MNYHEVTIIIKN------SSNNSLRKLGSYCV----LSIRETETRRFAAVCKLISILIK
allele_b        -MNYHEVTIIIKN------SSNNSLRKLGSYCV----LSIKETETRRFAAVCKLISILIK
SC              EQHQQPISIVFQNKKKKGDTSSPDIHTTLDFPLNGPHLCTHQFKLKRCAILLNLLKVVME
                  : : ::*:::*      :*. .:..  .: :    *. .: : .* * : :*:.::::

allele_a        HLKAQNQITTIRDIYYQDVEVFNHCQNECRFLLGQLVERGLGWSLCDDLNIHPTQKGLVY
allele_b        HLKAQNQVTTIRDIYYQDVEVFNHCQNECRFLLGQLVERGLGWSLCDDLNIHPTQKGLVY
SC              KLPLGKN-TTVRDIFYSNVELFQRQANVVQWL--DVIRFNFKLSPRKSLNIIPAQKGLVY
                :*   :: **:***:*.:**:*:.  *  .:*  :::  .:  *  ..*** *:******

allele_a        GDY----------------FQELSI-KAEPILIPINYTKFFNTKITQIKVEKVI-VVILE
allele_b        GDY----------------FQELSI-KAEPILIPINYTKFFNTKITQIKVEKVI-VVILE
SC              SPFPIDIYDNILTCENEPKMQKQTIFPGKPCLIPF----FQDDAVIKLGTTSMCNIVIVE
                . :                :*: :*  .:* ***:    * :  : :: . .:  :**:*

allele_a        KDAVFQCLCTHLRQHNIINRFLIVTAKGYSDNLTLRFLTWLQTN-----ENCKFIGFFDS
allele_b        KDAVFQCLCTHLRQHNIINRFLIVTAKGYSDNLTLRFLTWLQTN-----ENCKFIGFFDS
SC              KEAVFTKLVNNY--HKLSTNTMLITGKGFPDFLTRLFLKKLEQYCSKLISDCSI--FTDA
                *:***  * .:   *:: .. :::*.**:.* **  **..*:       .:*.:  * *:

allele_a        DVYGLNIYWQY---NQKLPEMV----YSGIYL---------LESQPHTWLSITLRDIAMM
allele_b        DVYGLNIYWQY---NQKLPEMV----YSGIYL---------LESQPHTWLSITLRDIAMM
SC              DPYGISIALNYTHSNERNAYICTMANYKGIRITQVLAQNNEVHNKSIQLLSLNQRDYSLA
                * **:.*  :*   *:. . :     *.** :         : .:.   **:. ** ::

allele_a        IKICKNGNNKYNDI---PYREL----TRGLYLFKKAEMNVVQANNANQSYVNYMVSKILG
allele_b        IKICKNGNNKYNDI---PYREL----TRGLYLFKKAEMNVVQANDANQSYVNYMVSKILG
SC              KNLIASLTANSWDIATSPLKNVIIECQREIFFQKKAEMNEIDA------------RIF-
                 ::  . . : .**   * .::     * ::: ****** ::*             .*:

allele_a        TPSYTK
allele_b        TASYTK
SC              --EYK-
                  .*.
```

Supplementary Figure 7. Alignment of Spo11 sequences. *S. cerevisiae* and *C. albicans* Spo11 alleles (a and b) were aligned with ClustalW and conserved positions are marked below. The catalytic aspartate is boxed in red.

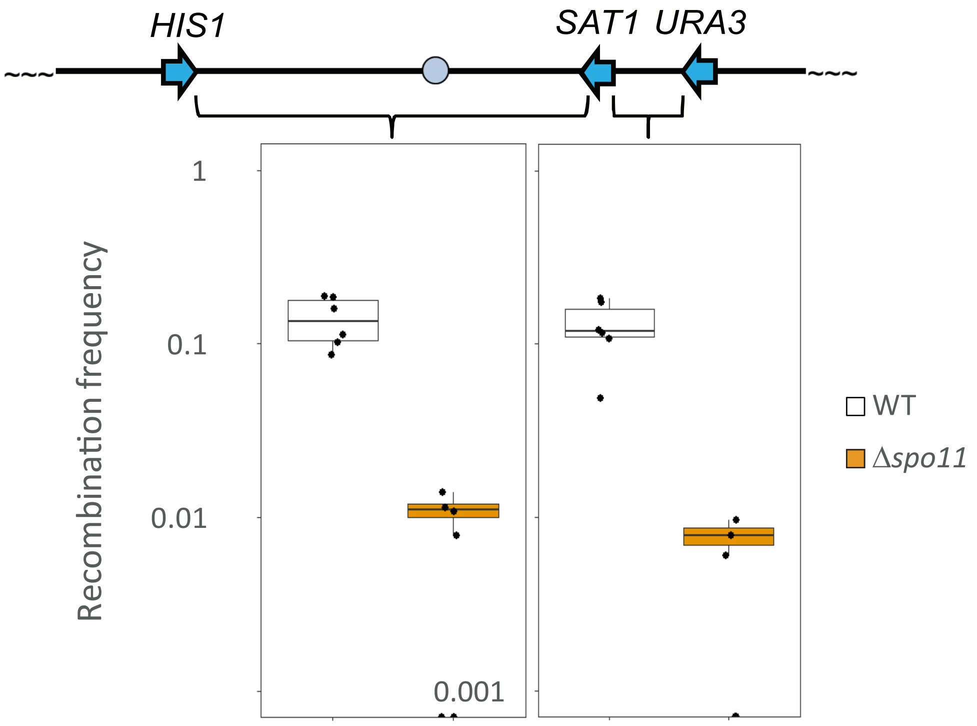

Supplementary Figure 8. Recombination during parasex in wildtype and Δ*spo11* strains. Recombination rates were measured across the *HIS1*-*SAT1* and *SAT1*-*URA3* intervals in the WT (white) and Δ*spo11* (orange) strain backgrounds. Boxplots are presented as the 75th to 25th percentile with the thick line denoting the median. Whiskers indicate the largest and smallest values within 1.5x of the interquartile range. *n* = 6 biologically independent experiments.

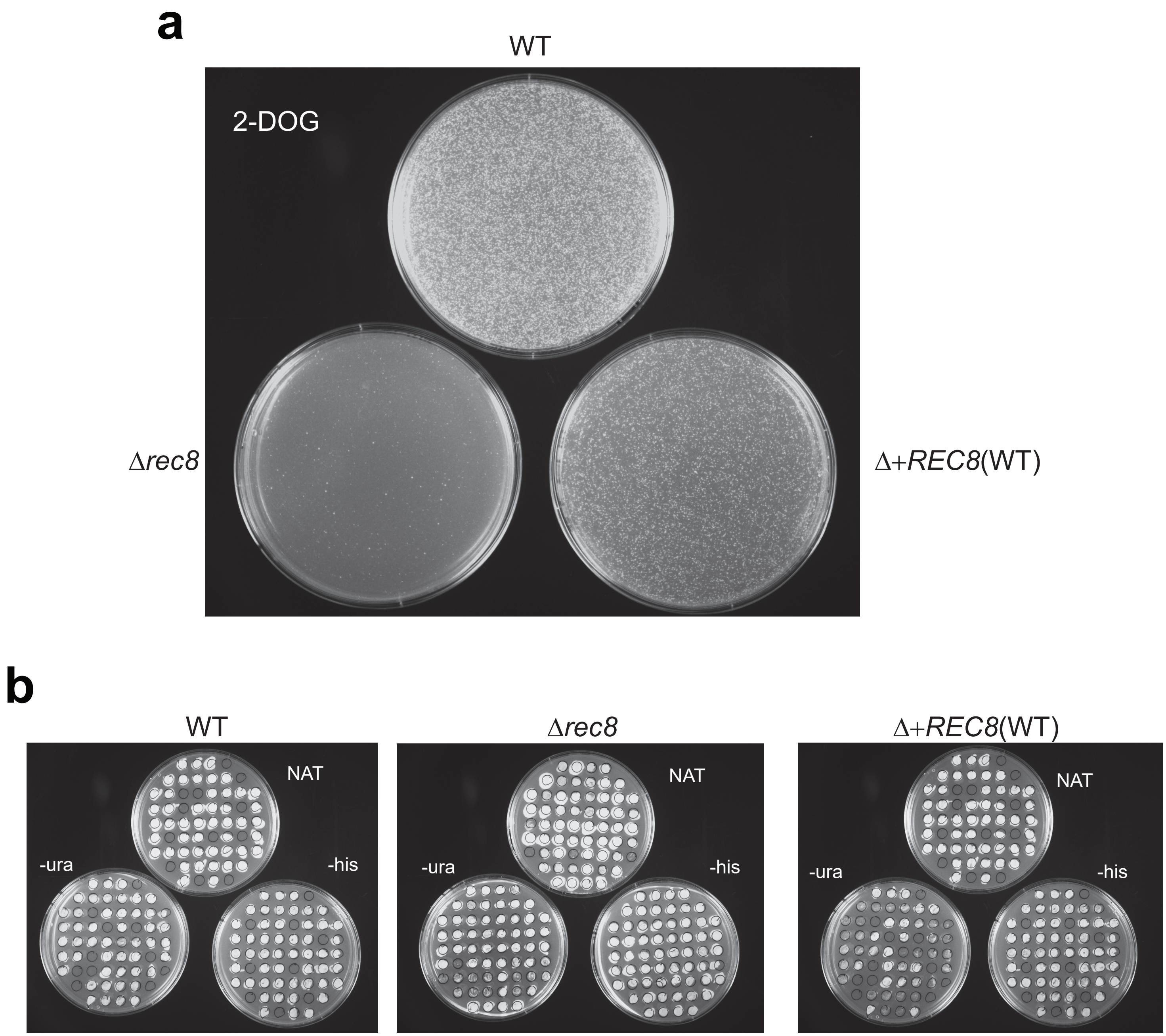

Supplementary Figure 9. ***REC8*** **increases chromosome instability and recombination under CCL conditions.** (**a**) Images of plates illustrating 2-DOG^R colonies produced by CCL in *C. albicans* wildtype and Δ*rec8* tetraploid strains, as well as in Δ*rec8* strains complemented with one *REC8* allele (Δ+*REC8*(WT)). (**b**) Images of plates examining CCL progeny of different *REC8* backgrounds grown on different selective media.

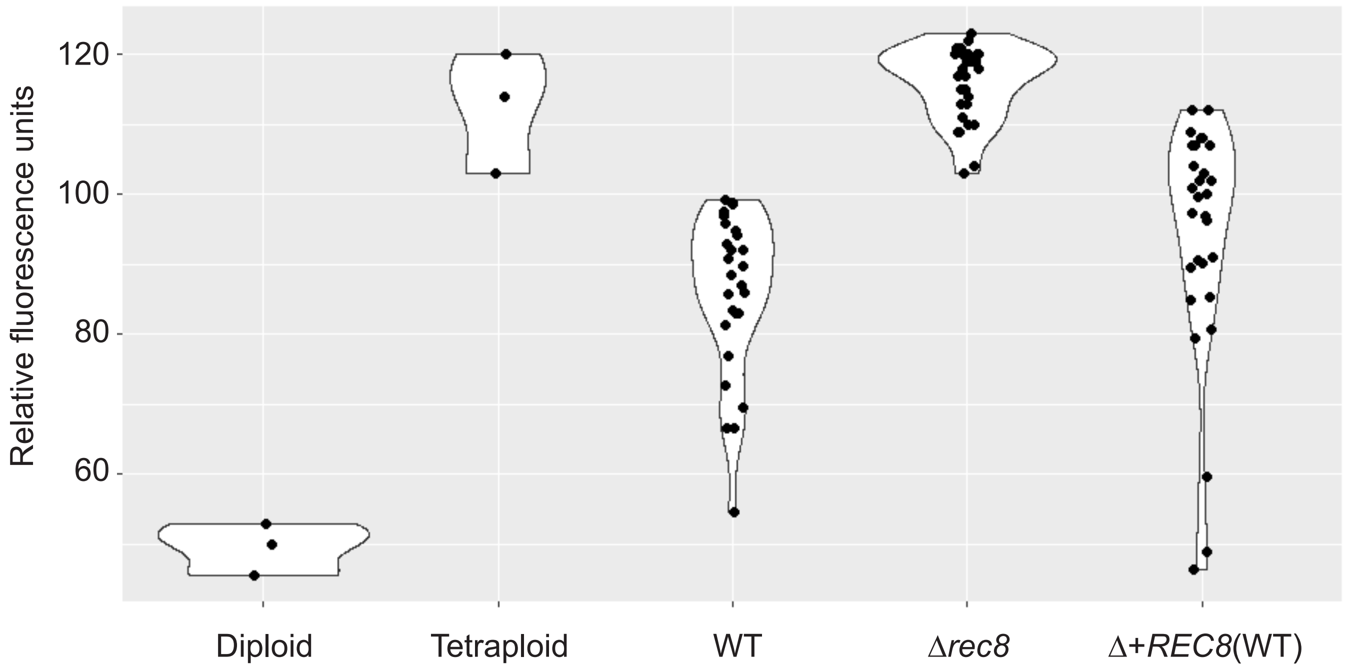

Supplementary Figure 10. DNA quantification of CCL products across *REC8* genotypes. Cells were cultured on PRE-SPO medium for 7 days at 37°C and then plated to single colonies on SCD medium. Thirty colonies from each plate were grown overnight in liquid YPD medium and the genomic content determined by flow cytometry of SYBR green-stained DNA. The relative fluorescence units were measured as the mean of the G1 peak. *n* = 28 biologically independent samples.

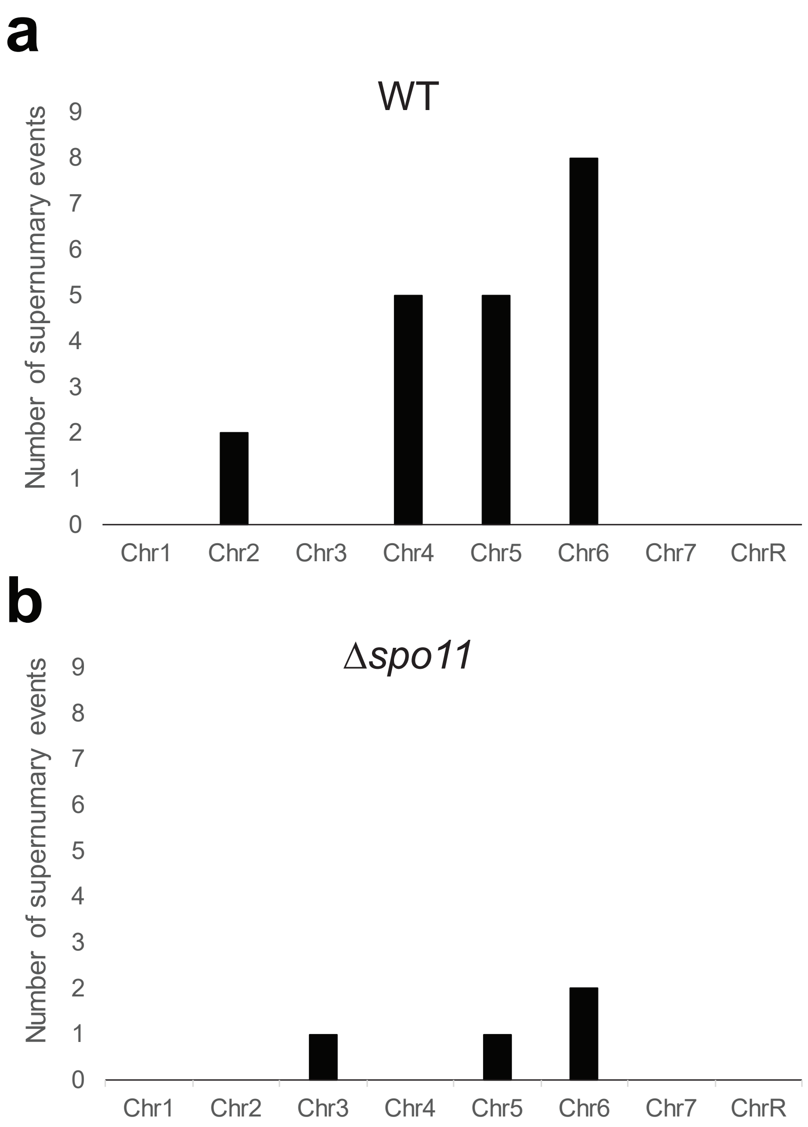

Supplementary Figure S11. Aneuploid chromosomes in WT and *SPO11* deletion strains. The frequency of trisomy for each chromosome among the 21 sequenced strains identified by ddRAD-Seq and YMAP[1] analysis are displayed for both the WT (**a**) and *SPO11* deletion (**b**) backgrounds.

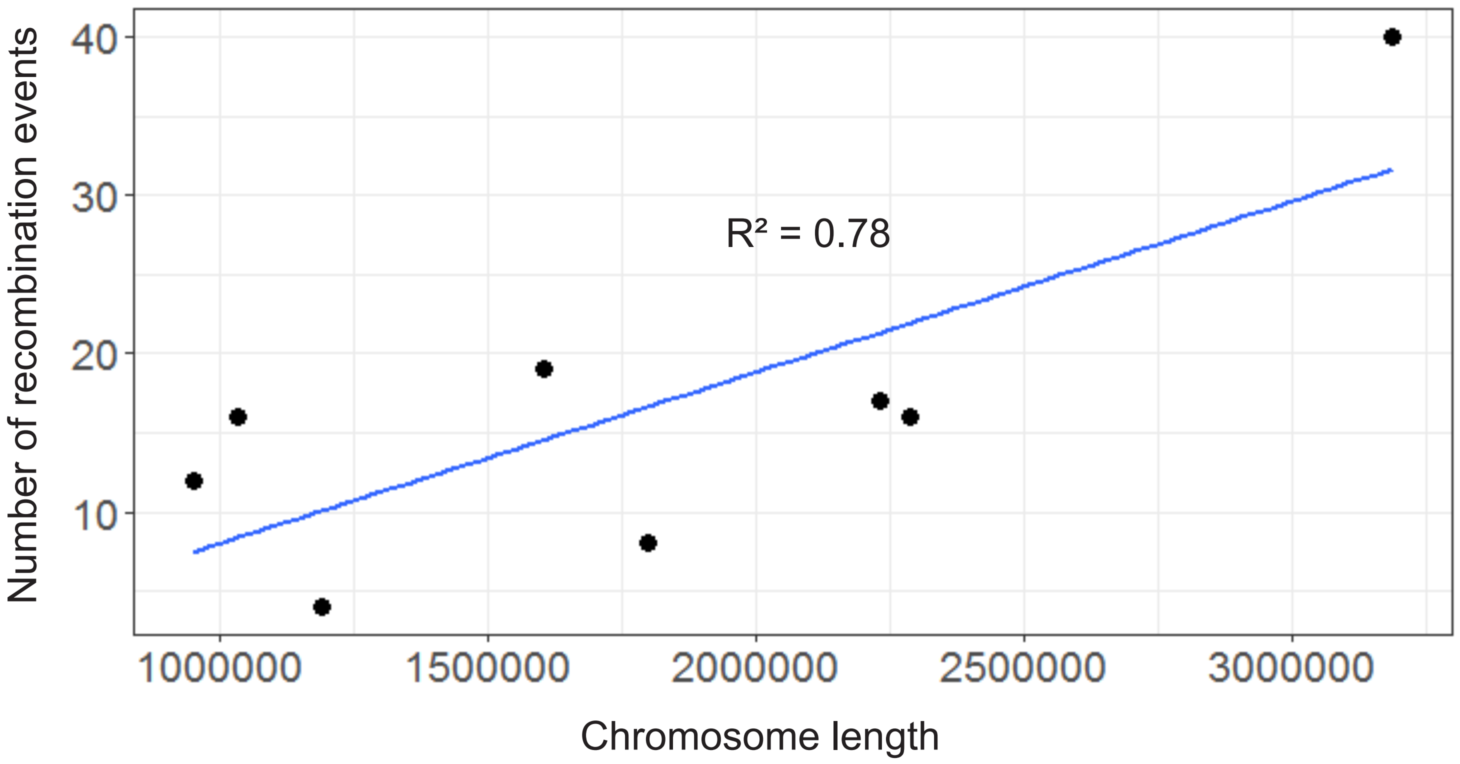

Supplementary Figure 12. Recombination events correlate with chromosome size. The number of recombination events identified by ddRAD-Seq in sequenced WT colonies were plotted against chromosome size. A linear best fit line (blue) is plotted for the data.

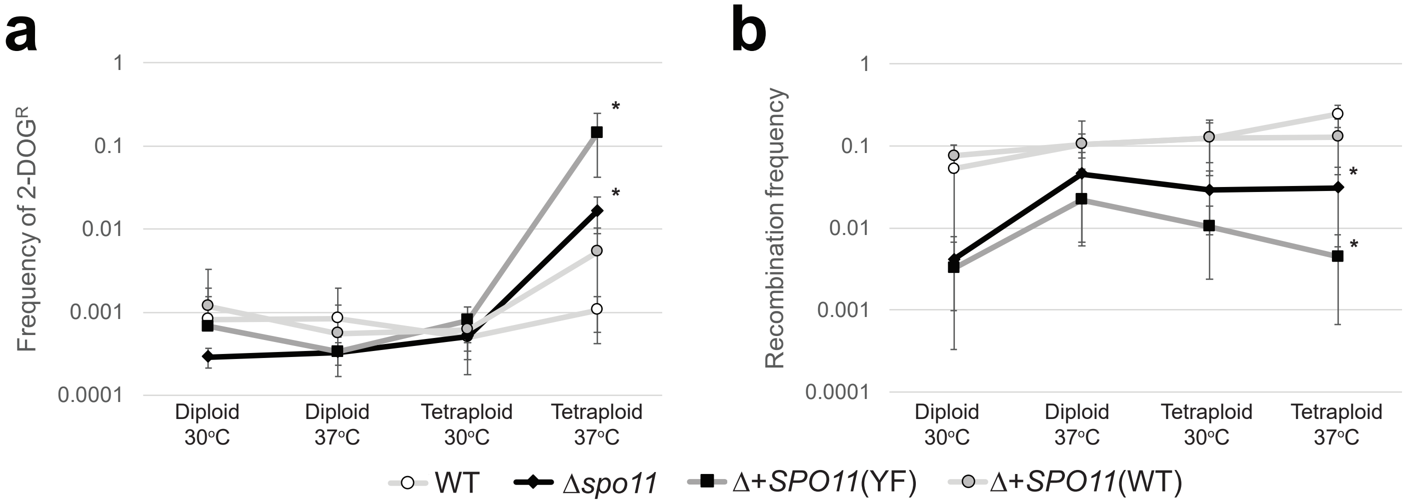

Supplementary Figure 13. Mitotic recombination frequencies are dependent on temperature, ploidy, and Spo11. The frequency of 2-DOG^R colonies (**a**) and recombination (**b**) is plotted for each genetic background across the different ploidy states, temperatures and genetic backgrounds during mitotic passaging. * denotes $p<0.05$ compared to the wildtype background as calculated by two-sample t (chromosome loss) or Wilcoxon (recombination frequency) tests. Error bars represent standard deviation. White, black, grey with black squares, and grey lines denote WT, Δ*spo11*, Δ+*SPO11*(YF), and Δ+*SPO11*(WT), respectively. *n* = 4, 5, 6, and 6 biologically independent experiments for 30°C diploid, 37°C diploid, 30°C tetraploid, and 37°C tetraploid, respectively. For recombination, *n* = 4, 4, 5, and 5 biologically independent experiments for 30°C diploid, 37°C diploid, 30°C tetraploid, and 37°C tetraploid, respectively.

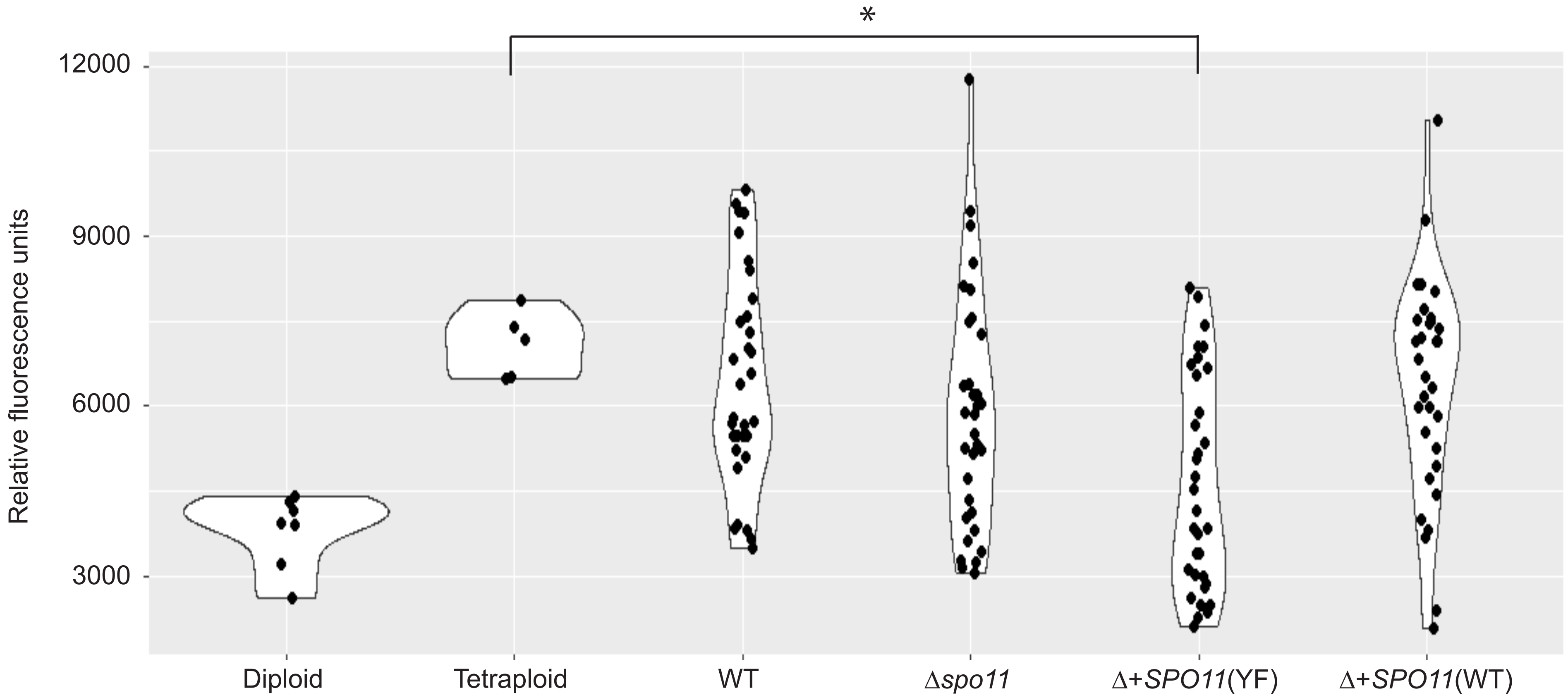

Supplementary Figure 14. DNA quantification of mitotically passaged strains with different *SPO11* genotypes. Tetraploid cells were serially passaged in YPD for 7 days at 30°C (cells diluted 1:100 every 24 h), and then plated to single colonies on SCD medium. Forty colonies from each plate were grown overnight in liquid YPD medium and the genomic content determined by flow cytometry of SYBR green-stained DNA. The relative fluorescent units were measured as the mean of the G1 peak. * denotes $p<0.05$ by Mann Whitney U test compared to the tetraploid. n = 33 biologically independent samples.

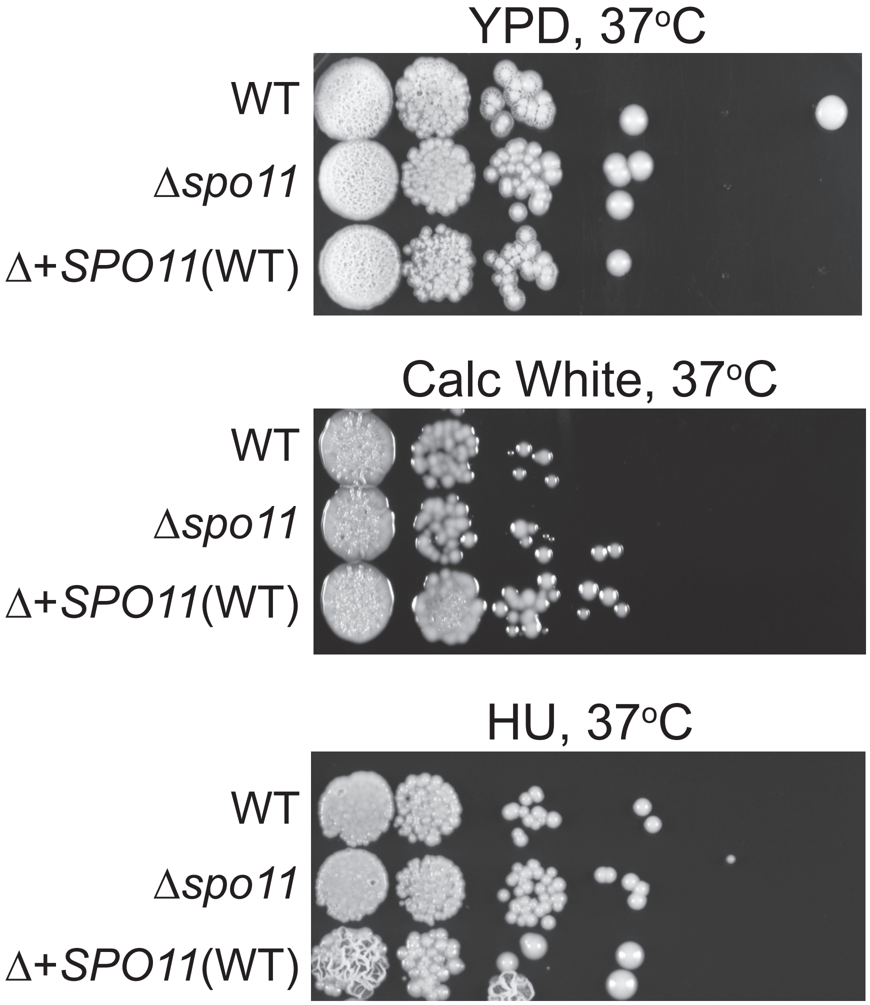

Supplementary Figure 15. *SPO11* shows a selective role in resistance to various stressors. Spot dilution assays were performed for wildtype, Δ*spo11* and Δ+*SPO11*(WT) tetraploid strains on SCD medium or medium containing 100 μg/mL calcofluor white or 2 mM hydroxyurea (HU) and imaged after 2 days at 37°C.

Supplementary references

1. Abbey, D.A. *et al.* YMAP: a pipeline for visualization of copy number variation and loss of heterozygosity in eukaryotic pathogens. *Genome Med* **6**, 100 (2014).
