# Supplementary material for: A ‘parameiosis’ drives depolyploidization and homologous recombination in Candida albicans
Source: Nat Commun. 2019 Sep 26;10:4388. doi: 10.1038/s41467-019-12376-2 (PMC6763455; doi:10.1038/s41467-019-12376-2)
Supplement: Supplementary file 3 — Reporting Summary [file 41467_2019_12376_MOESM3_ESM.pdf]

Corresponding author(s): Matthew Z Anderson
Richard J Bennett

Last updated by author(s): Aug 16, 2019

Reporting Summary

Nature Research wishes to improve the reproducibility of the work that we publish. This form provides structure for consistency and transparency in reporting. For further information on Nature Research policies, see Authors & Referees and the Editorial Policy Checklist.

Statistics

For all statistical analyses, confirm that the following items are present in the figure legend, table legend, main text, or Methods section.

n/a	Confirmed	
☐	☒	The exact sample size (*n*) for each experimental group/condition, given as a discrete number and unit of measurement
☐	☒	A statement on whether measurements were taken from distinct samples or whether the same sample was measured repeatedly
☐	☒	The statistical test(s) used AND whether they are one- or two-sided<br>*Only common tests should be described solely by name; describe more complex techniques in the Methods section.*
☒	☐	A description of all covariates tested
☐	☒	A description of any assumptions or corrections, such as tests of normality and adjustment for multiple comparisons
☐	☒	A full description of the statistical parameters including central tendency (e.g. means) or other basic estimates (e.g. regression coefficient) AND variation (e.g. standard deviation) or associated estimates of uncertainty (e.g. confidence intervals)
☐	☒	For null hypothesis testing, the test statistic (e.g. *F*, *t*, *r*) with confidence intervals, effect sizes, degrees of freedom and *P* value noted<br>*Give P values as exact values whenever suitable.*
☒	☐	For Bayesian analysis, information on the choice of priors and Markov chain Monte Carlo settings
☒	☐	For hierarchical and complex designs, identification of the appropriate level for tests and full reporting of outcomes
☒	☐	Estimates of effect sizes (e.g. Cohen's *d*, Pearson's *r*), indicating how they were calculated

Our web collection on statistics for biologists contains articles on many of the points above.

Software and code

Policy information about availability of computer code

Data collection	The only software used to collect data was the previously published pipeline called "YMAP" published by Judy Berman's lab (PMID 25505934).
Data analysis	All data analysis was performed in either Excel 2016 or R (version 3.3.1).

For manuscripts utilizing custom algorithms or software that are central to the research but not yet described in published literature, software must be made available to editors/reviewers. We strongly encourage code deposition in a community repository (e.g. GitHub). See the Nature Research guidelines for submitting code & software for further information.

Data

Policy information about availability of data

All manuscripts must include a data availability statement. This statement should provide the following information, where applicable:

- Accession codes, unique identifiers, or web links for publicly available datasets
- A list of figures that have associated raw data
- A description of any restrictions on data availability

The datasets generated during and/or analysed during the current study are available in this article and its Supplementary Information files, or from the corresponding author on reasonable request. Sequence data used for ddRAD-Seq analysis is available at the Sequence Read Archive (SRA; https://submit.ncbi.nlm.nih.gov/subs/sra/) under the submission SUB6179990 ([http://www.ncbi.nlm.nih.gov/bioproject/560397]).

Field-specific reporting

Please select the one below that is the best fit for your research. If you are not sure, read the appropriate sections before making your selection.

☒ Life sciences ☐ Behavioural & social sciences ☐ Ecological, evolutionary & environmental sciences

For a reference copy of the document with all sections, see nature.com/documents/nr-reporting-summary-flat.pdf

Life sciences study design

All studies must disclose on these points even when the disclosure is negative.

Sample size	Sample sizes were determined using a predetermined number of experimental repeats of five expected experiments for main experiments. Three experiments were typically performed for supporting experiments.
Data exclusions	Data was not excluded from analysis. Overcrowding of some plates required their omission due to an inability to quantify colonies but no data able to be collected was excluded.
Replication	The experiments described within this manuscript were performed across two labs (The Bennett and Anderson labs) at two different institutions by multiple lab members to insure consistency.
Randomization	All samples for experimental sets (WT, mutants, and complemented strains) were performed together. Individual experiments were performed on independent days.
Blinding	Blinding was used by coding individual samples within experiments. A key was later used to decode samples.

Reporting for specific materials, systems and methods

We require information from authors about some types of materials, experimental systems and methods used in many studies. Here, indicate whether each material, system or method listed is relevant to your study. If you are not sure if a list item applies to your research, read the appropriate section before selecting a response.

Materials & experimental systems

n/a	Involved in the study
☐	☒ Antibodies
☐	☒ Eukaryotic cell lines
☒	☐ Palaeontology
☒	☐ Animals and other organisms
☒	☐ Human research participants
☒	☐ Clinical data

Methods

n/a	Involved in the study
☒	☐ ChIP-seq
☐	☒ Flow cytometry
☒	☐ MRI-based neuroimaging

Antibodies

Antibodies used	mouse anti-PSTAIR (Abcam, ab10345, https://www.abcam.com/pstair-antibody-pstair-ab10345.html), mouse anti-GFP polyclonal (Sigma, 11814460001, https://www.sigmaaldrich.com/catalog/product/roche/11814460001?lang=en&region=US)
Validation	The anti-PSTAIR antibody website notes validation in a series of lysates and loss following TEV cleavage (file:///C:/Users/anderson.3196/Downloads/datasheet_10345.pdf) in addition to validation data provided on the antibody main site (https://www.abcam.com/pstair-antibody-pstair-ab10345.html). The mouse anti-GFP antibody documentation notes validation using GFP+ and GFP- cell lines (https://www.sigmaaldrich.com/content/dam/sigma-aldrich/docs/Roche/Bulletin/1/11814460001bul.pdf).

Eukaryotic cell lines

Policy information about cell lines

Cell line source(s)	SC5314 Candida albicans
Authentication	The isolate had been sequenced as part of work coincident with this work. Additional sequencing performed here by ddRAD-Seq verified the strain.
Mycoplasma contamination	Mycoplasma is not a factor in yeast studies.

Commonly misidentified lines (See ICLAC register)	*Name any commonly misidentified cell lines used in the study and provide a rationale for their use.*

Flow Cytometry

Plots

Confirm that:

- [x] The axis labels state the marker and fluorochrome used (e.g. CD4-FITC).
- [x] The axis scales are clearly visible. Include numbers along axes only for bottom left plot of group (a 'group' is an analysis of identical markers).
- [ ] All plots are contour plots with outliers or pseudocolor plots.
- [ ] A numerical value for number of cells or percentage (with statistics) is provided.

Methodology

Sample preparation	Briefly, cells were washed and resuspended in 50:50 TE (50mM Tris pH 8/50mM EDTA) solution. Cells were then treated with 1mg/mL RNAse A for four hours at 37oC followed by 5mg/mL Proteinase K treatment at 37oC for 45 minutes. Cells were washed with 50:50 TE and resuspended in SybrGreen (1:100 dilution in 50:50 TE) and incubated overnight at 4oC. Stained cells were washed and resuspended in 50:50 TE and SybrGreen staining data was obtained for 50,000 events for each sample.
Instrument	BD FACSCanto II
Software	FlowJo v7 was used for analysis.
Cell population abundance	All samples were composed of ~99% yeast cells and did not require significant fractionation as negative controls contained less than 10 events.
Gating strategy	All events were included in the assessment of SYBR Green intensity as a measure of DNA content.

- [ ] Tick this box to confirm that a figure exemplifying the gating strategy is provided in the Supplementary Information.
